# Discovery of an Abundant Viral Genus in Polar Regions through the Isolation and Genomic Characterization of a New Virus against *Oceanospirillaceae*

**DOI:** 10.1128/aem.01896-22

**Published:** 2023-03-28

**Authors:** Wenjing Zhang, Yundan Liu, Kaiyang Zheng, Jinyan Xing, Qian Li, Chengxiang Gu, Ziyue Wang, Hongbing Shao, Cui Guo, Hui He, Hualong Wang, Yeong Yik Sung, Wen Jye Mok, Li Lian Wong, Yantao Liang, Andrew McMinn, Min Wang

**Affiliations:** a College of Marine Life Sciences, Institute of Evolution and Marine Biodiversity, Frontiers Science Center for Deep Ocean Multispheres and Earth System, Center for Ocean Carbon Neutrality, Ocean University of China, Qingdao, China; b School of Oceanography, Shanghai Jiao Tong University, Shanghai, China; c The Affiliated Hospital of Qingdao University, Qingdao, China; d UMT-OUC Joint Centre for Marine Studies, Qingdao, China; e Institute of Marine Biotechnology, Universiti Malaysia Terengganu (UMT), Kuala Nerus, Malaysia; f Institute for Marine and Antarctic Studies, University of Tasmania, Hobart, Tasmania, Australia; g Haide College, Ocean University of China, Qingdao, China; University of Nebraska-Lincoln

**Keywords:** *Oceanospirillum* phage vB_OsaM_PD0307, *Oceanospimyovirus*, genomic and phylogenetic analysis, ecological distribution

## Abstract

The marine bacterial family *Oceanospirillaceae*, is well-known for its ability to degrade hydrocarbons and for its close association with algal blooms. However, only a few *Oceanospirillaceae*-infecting phages have been reported thus far. Here, we report on a novel *Oceanospirillum* phage, namely, vB_OsaM_PD0307, which has a 44,421 bp linear dsDNA genome and is the first myovirus infecting *Oceanospirillaceae*. A genomic analysis demonstrated that vB_OsaM_PD0307 is a variant of current phage isolates from the NCBI data set but that it has similar genomic features to two high-quality, uncultured viral genomes identified from marine metagenomes. Hence, we propose that vB_OsaM_PD0307 can be classified as the type phage of a new genus, designated *Oceanospimyovirus*. Additionally, metagenomic read mapping results have further shown that *Oceanospimyovirus* species are widespread in the global ocean, display distinct biogeographic distributions, and are abundant in polar regions. In summary, our findings expand the current understanding of the genomic characteristics, phylogenetic diversity, and distribution of *Oceanospimyovirus* phages.

**IMPORTANCE** *Oceanospirillum* phage vB_OsaM_PD0307 is the first myovirus found to infect *Oceanospirillaceae*, and it represents a novel abundant viral genus in polar regions. This study provides insights into the genomic, phylogenetic, and ecological characteristics of the new viral genus, namely *Oceanospimyovirus*.

## INTRODUCTION

Viruses are the most abundant and diverse “life forms” in the ocean, and they mediate the mortality of marine microorganisms and biogeochemical cycles (1–3). Viruses that infect bacteria, named phages, also promote the active evolution and shape the phylogeny of their hosts through horizontal gene transfer (4, 5). Over the past few decades, our understanding of viral diversity has significantly expanded, mostly through the development of high-throughput sequencing and metagenomic technology (6–8). As a consequence, over 90% of available viral genomes remain uncultured and uncharacterized (hereinafter referred to as uncultivated viral genomes [UViGs]) (7). The targeted phage isolation methods are vital in terms of filling the knowledge gaps between sequence information and functionality, especially in the context of interaction dynamics between a phage and its host, as well as in shedding light on their potential role in marine microbial food webs.

*Oceanospirillaceae*, a bacterial family within the order Oceanospirillales, currently consists of 26 genera, and the first member of the lineage was isolated from coastal waters in 1957 (9). Most genera in this family are strictly aerobic and are either halotolerant or halophilic (10, 11). They require sodium ions for growth and are widely distributed in marine environments. Although their abundance is usually low, they are often dominant in specific microbial niches, such as the mucus of hermatypic corals and copepods (12, 13), algal bloom events in polar regions (14–16), oil-contaminated marine environments (17–19), and the Mariana Trench (20). This family is also well-known for its ability to degrade hydrocarbons (21–23) and for having complex relationships with algal blooms (14–16, 24).

Currently, only eight cultivated phages infecting *Oceanospirillaceae* strains have been reported, two of which have not yet been classified, and the remaining strains have been classified into *Autographiviridae* (three phages), *Siphoviridae* (two phages), and *Corticoviridae* (one phage) (18–20). However, myoviruses infecting *Oceanospirillaceae* have not previously been reported (25–27).

Here, we report on the isolation and genomic characterization of the first myovirus infecting *Oceanospirillaceae*, named vB_OsaM_PD0307. The genome of vB_OsaM_PD0307 shows large variance from the other isolated phages in the current NCBI data set, but it presents interesting, similar features with two UViGs that were assembled from marine metagenomes. So, we propose the creation of the new genus *Oceanospimyovirus*, featuring phage vB_OsaM_PD0307 as the type species. In addition, we undertook a genomic analysis and investigated the relative abundance of this new viral genus, and we have provided an insight into the genomic diversity, phylogenetic evolution, and biogeographic distribution patterns of this genus.

## RESULTS AND DISCUSSION

### Isolation and characterization of host and phage strains.

Phage vB_OsaM_PD0307 and its propagated host *Oceanospirillum* sp. PD0307 were isolated from surface seawaters of the Yellow Sea. According to the phylogenetic tree of 16S rRNA, the closest related bacterial strain is Oceanospirillum sanctuarii AK56, but they have a shared similarity of only 94.78% (Fig. S1). Based on the threshold value of 98.65% similarity in the 16S rRNA gene sequences for bacterial species delineation (28), *Oceanospirillum* sp. PD0307 is a novel species of the *Oceanospirillum* genus.

**FIG 1:**
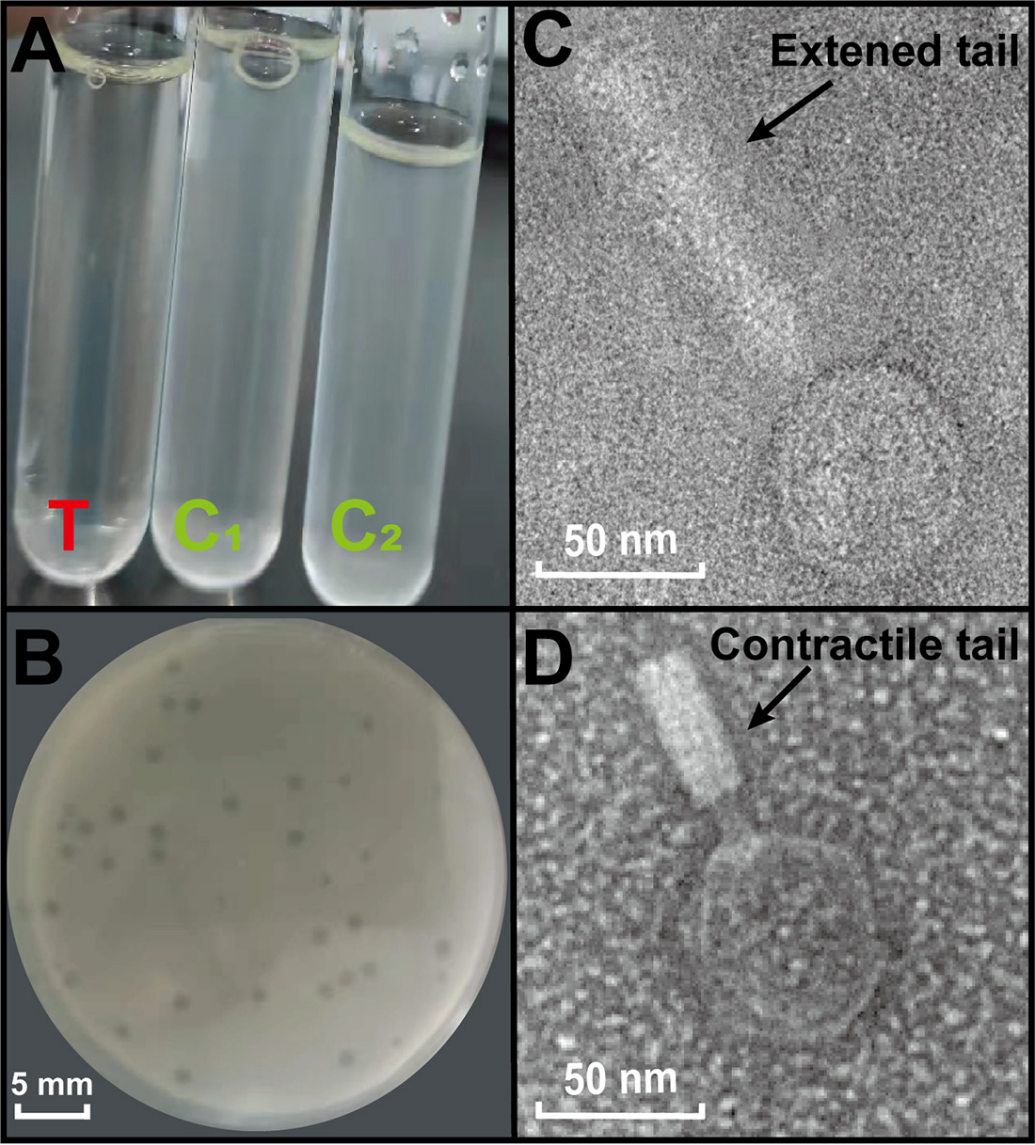
(A) The host strain was incubated with phage vB_OsaM_PD0307 suspension for 24 h. T, treatment; C1, control 1; C2, control 2. (B) Plaques formed in double-layer agar plates. The scale bar is 5 mm. (C) Transmission electron microscopy of phage vB_OsaM_PD0307. The scale bar is 50 nm.

Phage vB_OsaM_PD0307 formed clear and round (1 to 2 mm diameter average) plaques on the bacterial lawns. Unlike the untreated groups, the host bacterial suspension became clear after incubation with *Oceanospirillum* phage vB_OsaM_PD0307, which strongly indicates that the bacteria were killed by the phages (Fig. 1A and B). In addition to the experimental methods, the bioinformatic software package BACPHLIP (29), which is a phage lifestyle predictor, predicated that vB_OsaM_PD0307 has a high virulence possibility. The morphology of vB_OsaM_PD0307 has the characteristics of a myovirus with an icosahedral head of 51 ± 2 nm in diameter and a 112 ± 3 nm extended tail (Fig. 1C).

### Phage packaging strategy and termini mechanisms.

The genome of phage vB_OsaM_PD0307 was assembled to be 45,805 bp. Of the total 283 Mb raw sequence reads, 97% mapped to the contig. The packaging strategy and termini mechanisms were reported by utilizing the PhageTerm software package (30). The analysis revealed the presence of redundant DNA ends with exact 496-bp repeats, indicating that it represents the complete phage genome. The analysis report of PhageTerm revealed that vB_OsaM_PD0307 uses the phage P1-type headful packaging mechanism, starting from a pac site (Fig. S2). The phage terminase initiates the packaging of the genome concatemer at the specific pac site, and its packaging is terminated at variable positions when the phage head becomes full (30). The facts that packaging is directional, that no precise cut is made upon the termination of packaging, and that a peak is expected only in a single orientation suggest that vB_OsaM_PD0307 has a forward packaging direction.

### Phage genome annotation.

**FIG 2:**
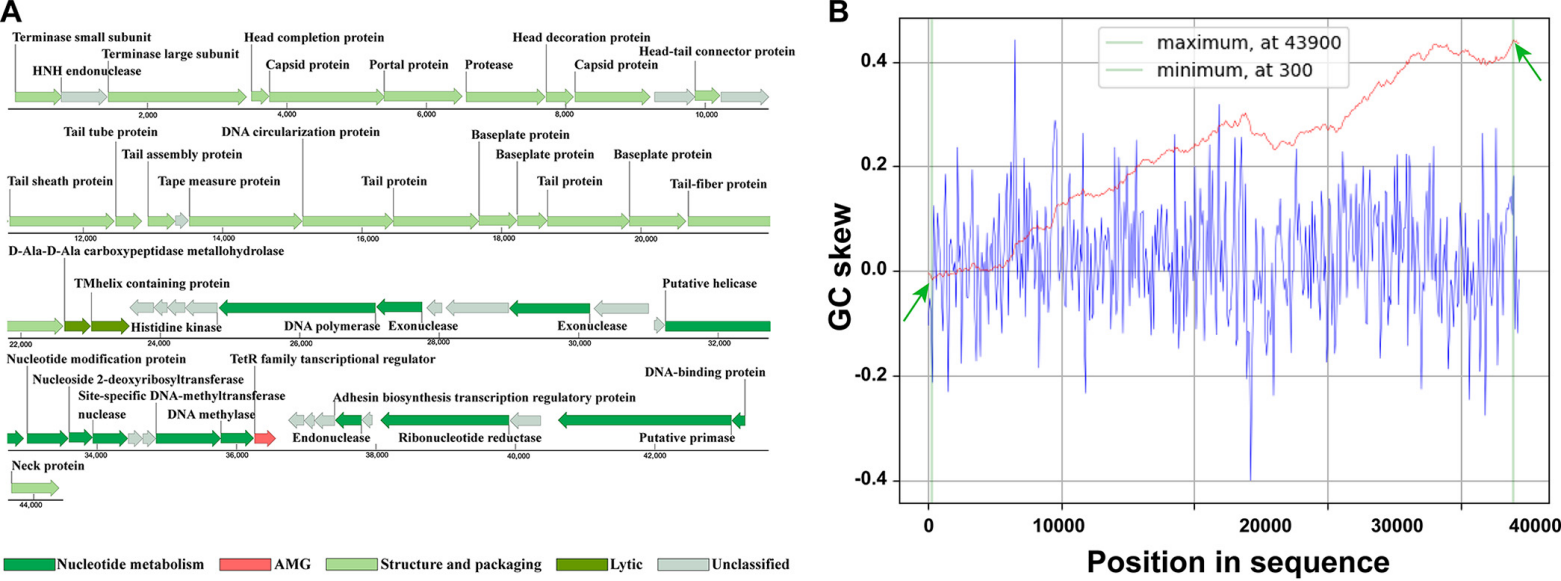
Genome map and cumulative GC skew analysis of phage vB_OsaM_PD0307. (A) ORFs are represented by arrows, with the arrow points indicating the directions of their transcription. They are marked using distinct colors, according to their functions. (B) The global minimum and maximum are displayed in the cumulative graph and were calculated by using a window size of 100 bp and a step size of 100 bp. The GC-skew and the cumulative GC-skew are represented by blue and red lines, respectively. The minimum and maximum of the GC-skew are indicated by two green arrows.

According to the genomic sequencing and assembly results, the phage vB_OsaM_PD0307 has a 44,421-bp linear dsDNA genome with a GC content of 57.13%. The cumulative GC skew shows the predicted origin of replication and replication terminus located at 300 nt and 43,900 nt, respectively (Fig. 2B). In the phage genome, 37 genes are located on the sense strand, and 19 genes are located on the antisense strand, consisting of a large proportion of orphan or unclassified genes (33.92%). All 56 genes could be classified into three different modules, except for two lysis genes and one auxiliary metabolic gene (AMG), based on the functions of homologous sequences and detected conserved domains (Fig. 2A; Table S1).

The genome of vB_OsaM_PD0307 encodes 13 predicted open reading frames (ORFs) that are putatively involved in nucleotide metabolism. ORF 44 and ORF 45 encoded DNA-methyltransferase and DNA methylase, respectively. In prokaryotes, the methylation of native DNA acts as a type of restriction-modification (R-M) system and prevents endogenous DNA from being degraded by restriction endonucleases (31). Most foreign DNA lacks the appropriate methylation profile and is therefore cleaved by the restriction endonuclease activities of the bacterium’s R-M systems. However, many phage genomes have also been found to contain methylases, methyltransferases, etc. (32–35), with the major role of methylation in the phage genome being to counteract host-encoded restriction systems (36).

21 proteins related to the structure and packaging modules are located upstream of the genome. The putative protease (ORF 7) is affiliated with the cl23717 superfamily, which has portal proteins upstream and capsid proteins downstream. Capsid maturation in double-stranded-DNA (dsDNA) phages requires proteolytic cleavage by a prohead protease (37). In the BLAST results, ClpP/crotonase-like domain proteins were significantly matched to the S49 family proteins, which are members of the large crotonase superfamily (38). One of the typical features of myovirus is the presence of tail sheath genes, and the ORF 13 of phage vB_OsaM_PD0307 encoded this gene. This is highly homologous to the tail sheath gene of *Shewanella* phage SppYZU01, which is also a myovirus. The top 50 homologous sequences are all present in the myovirus under the given thresholds (E value < 1×10^−50^, qcov > 90%, pident > 30%).

ORF 25 and ORF 26 may be responsible for viral lysis. ORF25 represents a C-terminal domain of zinc D-Ala-D-Ala carboxypeptidases (subfamily M15A, PF08291.14), which catalyzes carboxypeptidation but not transpeptidation reactions that are involved in bacterial cell wall metabolism (39–41). d-Ala-d-Ala metallopeptidases have similar active sites and share a core folding motif with lysostaphin-type peptidases. The central Zn^2+^ is tetrahedrally coordinated by two histidines, an aspartate, and a water molecule. The same arrangement of active-site residues is also found in lysostaphin-type enzymes (42). The TMhelix-containing protein (ORF 26) is affiliated with the family of bacterial 3TM holins (PF11351.11). The protein in Rhodobacter capsulatus is expressed overlapping and downstream of an endolysin, N-acetylmuramidase lysozyme (43, 44). It is thought that the combined actions of endolysins and holins are responsible for the phage particles that were released from the host cell.

The *in silico* prediction annotation process identified an important AMG, namely, ORF 46, which encodes the *TetR* family transcriptional regulator. It is homologous to the same family protein as *Haliea* sp. (E value 1×10^−15^, qcover 96%, pident 43.43%, accession RZJ26198.1) and contains a conserved domain, namely TetR_N (pfam00440), which has been detected in it. The *TetR* (tetracycline repressor proteins) family of regulators is a large and important family of one-component systems in prokaryotic signal transduction mechanisms (45–47). The members of this family are best known for their roles in conferring antibiotic resistance to large categories of bacterial species. *TetR* represses the expression of *TetA*, which is a membrane protein that pumps out substances that are toxic to the bacteria, such as tetracycline, by binding the *TetA* operator (45, 48). Since phages require active host cells to complete lysis (49), it is speculated that this gene in phages could assist bacteria in surviving environmental antibiotics in order to successfully infect and finally contribute to its proliferation success in marine environments. Because there are many unknown protein functions in the vB_OsaM_PD0307 phage genome, it is thought that with the continuous improvement of the protein function database, other interesting AMGs will be discovered.

### vB_OsaM_PD0307 is the type phage of the proposed genus *Oceanospimyovirus*.

Currently, eight phages that infect *Oceanospirillaceae* have been isolated (Table 1), but vB_OsaM_PD0307 is the first myovirus that infects *Oceanospirillaceae*. Genomic and phylogenetic comparisons between vB_OsaM_PD0307 and previously described phages show marked differences, not only morphologically but also in genomic features (Fig. S3).

**TABLE 1:** The information of all isolated phages against *Oceanospirillaceae*

Species	Family	Accession	No. of ORF	Genome size (bp)	GC content	Host strain
*Oceanospirillum* phage vB_OsaM_PD0307	*Myoviridae*	OL658619	56	44,421	57.13%	Oceanospirillum sanctuarii
*Oceanospirillum* phage vB_OliS_GJ44	*Siphoviridae*	MW560978	60	33,786	48.77%	Oceanospirillum sp. ZY01
*Marinomonas* phage MfV	Unclassified	MW618650	20	10,075	46.01%	*Marinomonas*
*Marinomonas* phage YY	*Corticoviridae*	MH105080	4	8,828	57.05%	*Marinomonas* sp.
*Marinomonas* phage CB5A	*Autographiviridae*	MF481197	54	45,385	43.22%	Marinomonas mediterranea
*Marinomonas* phage CPP1m	*Autographiviridae*	KY626176	50	44,245	43.36%	Marinomonas mediterranea
*Marinomonas* phage CPG1g	*Autographiviridae*	KY626177	50	44,244	43.36%	Marinomonas mediterranea
*Nitrincola* phage 1M3-16	Unclassified	KJ534580	144	82,438	41.31%	*Nitrincola* sp. 1M3-6
*Marinomonas* phage P12026	*Siphoviridae*	JQ867100	54	31,766	46.60%	Marinomonas pontica IMCC12026

To identify the exact taxon of the phage vB_OsaM_PD0307, a proteomic tree of viral genome sequences was established by using ViPtree with all of the reference viruses in the Virus-Host DB (50, 51). The tree shows that vB_OsaM_PD0307 represents a separate cluster that is far from other isolated phages (Fig. S4). Subsequently, a second phylogenetic tree that included not only the GenBank isolates but also UViGs as a reference data set was constructed. The data set included all of the isolated Caudovirales in the GenBank database and all of the uncultivated UViGs (more than two million) in the IMG/VR v3 database (52). Finally, 595 related viral sequences (192 RefSeq from NCBI, 403 UViGs from IMG/VR) with an average weight of 30.11 were recruited and grouped using vConTACT 2 (Fig. S5) (53). There are two UViGs (Station137_MES_COMBINED_FINAL_NODE_1159_length_42346_cov_88.299047, hereinafter referred to as S137_MES_NODE_1159 and Station85_DCM_COMBINED_FINAL_NODE_526_length_43166_cov_67.870497, hereinafter referred to as S85_DCM_NODE_526) that showed high weights (>100) with vB_OsaM_PD0307, and all three of them belong to the same viral cluster (VC_113). The average nucleotide identities (ANIs) among them are between 76.01% and 85.27% (Fig. 5B). Under the current guidelines of The International Committee on the Taxonomy of Viruses (ICTV), new genera are genomes that have an ANI of ≥70% to the type species of a particular genus (54). As such, we have proposed that the three phages be considered members of a new genus called *Oceanospimyovirus*, featuring phage vB_OsaM_PD0307 as the type species.

The water samples that were assembled to S85_DCM_NODE_526 and S137_MES_NODE_1159 were collected from the sea. Their virulence possibilities were 93.75% and 76.25%, respectively. BACPHLIP predicted phage vB_OsaM_PD0307 to have a lytic lifestyle, and infection experiments also demonstrated this. Two homologous UViGs, members of *Oceanospimyovirus*, may also have similar lifestyles. (29) The potential host of S137_MES_NODE_1159 was *Roseobacteraceae*, *Mameliella*, which shows the wide host diversity of *Oceanospimyovirus* (Table 2).

**TABLE 2:** The features of *Oceanomyovirus*

Feature	vB_OsaM_PD0307	S85_DCM_NODE_526	S137_MES_NODE_1159
Sample collection	Marine	Marine	Marine
Sequence resource	Isolated	UViG (IMG/VR)	UViG (IMG/VR)
Genome topology	Linear contig	Linear contig	Linear contig
Genome size (bp)	44421	43166	42346
Genome completeness	100%	92.17%	94.47%
GC content	57.13%	56.44%	58.86%
Virulent possibility	70.00%	93.75%	76.25%
Taxon	*Myoviridae*	Unclassified	Unclassified
Host	*Oceanospirillum* (isolated)	Unclassified	*Mameliella* (predicted)

**FIG 3:**
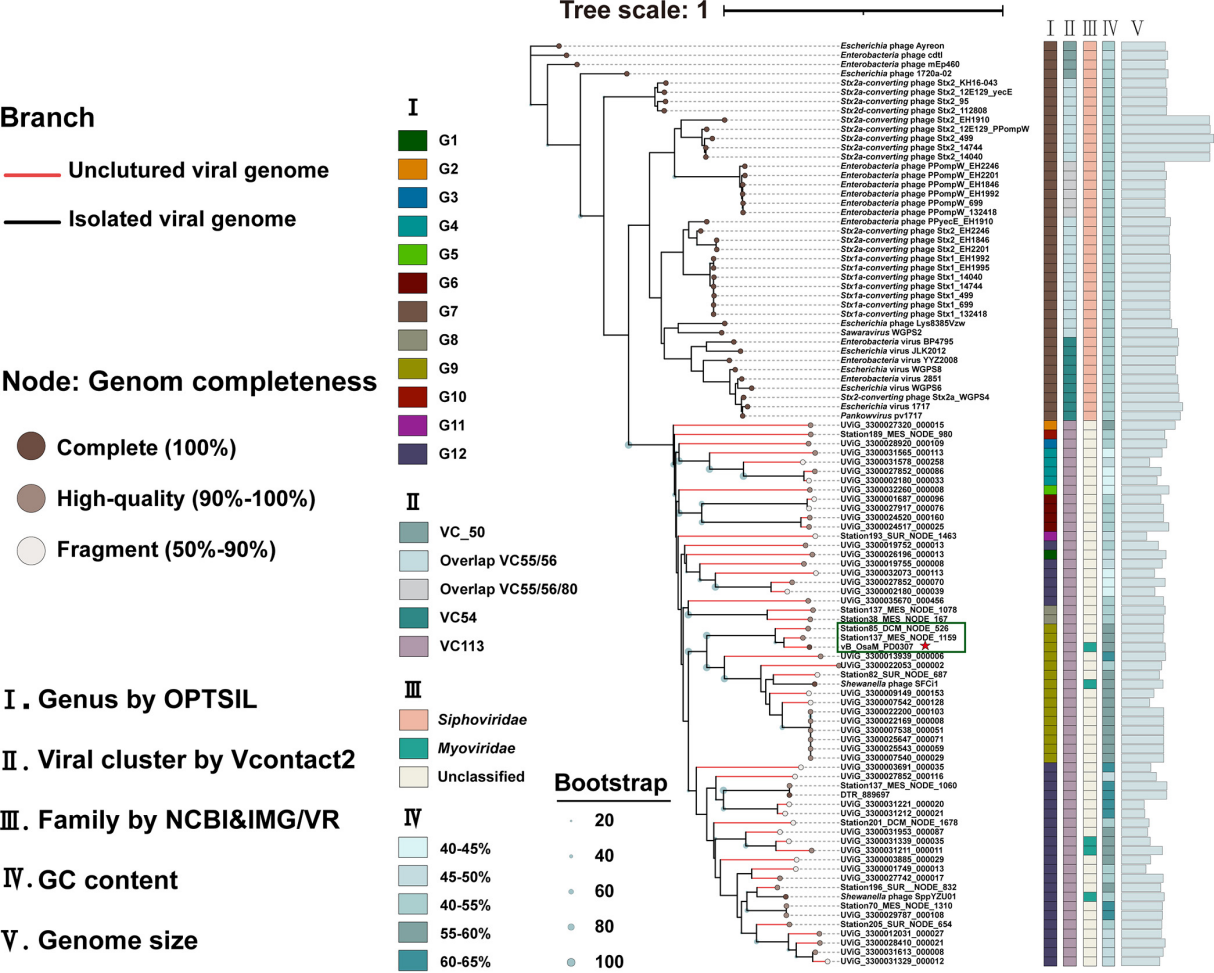
Whole-genome-based phylogenetic tree of vB_OsaM_PD0307 and related viral sequences. The whole-genome-based phylogenetic tree was constructed using VICTOR with the formula D6. The VC and OPTSIL clusters are at the genus levels. Each genus is indicated by a unique color. The minimum and maximum genome sizes are 23,167 bp and 93,786 bp, respectively. vB_OsaM_PD0307 is shown with a red star in the green box.

**FIG 4:**
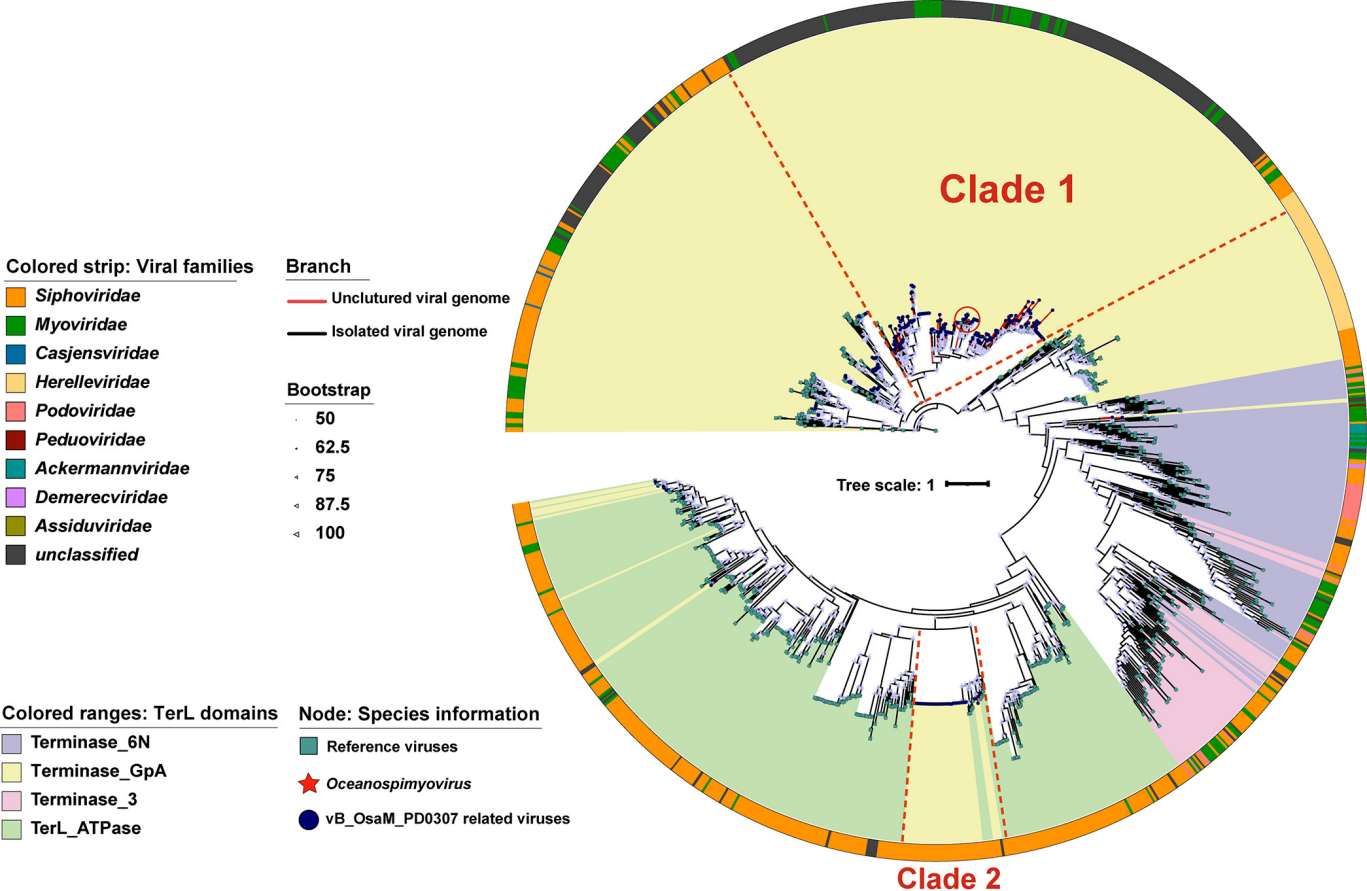
Phylogenetic analysis of the vB_OsaM_PD0307 TerL domain. A maximum-likelihood tree was constructed using TerL domain sequences. The vB_OsaM_PD0307-related phages were mainly grouped into two clades, based on phylogeny. Shading is used to indicate the TerL domains. All three members of *Oceanospimyovirus* are represented with red stars in the red circle. The reference viral TerL domain sequences are indicated by a green rectangle. The scale bar indicates the number of amino acid substitutions per site.

A whole-genome phylogenetic tree that was inferred using the Genome-BLAST Distance Phylogeny method (GBDP) and the OPTSIL taxon through VICTOR found that vB_OsaM_PD0307 (55), S137_MES_NODE_1159, and S85_DCM_NODE_526 were on the same branch, clustered together (shown in the green box) (Fig. 3), and belonged to the identified genus, namely, *Oceanospimyovirus* (Fig. 3). Another phylogenetic tree of terminase large subunits (TerL) domains was constructed according to the vConTACT results, and it provided further support for this proposal (Fig. 4). The TerL domains of all 391 vB_OsaM_PD0307 related viruses (291 UViGs, 100 isolates) were Terminase_GPA type (PF05876) and were mainly classified into two clades. 95.5% of the UViGs are mostly concentrated in clade 1, which has been shown to be monophyletic.

### Synteny and motif analysis.

**FIG 5:**
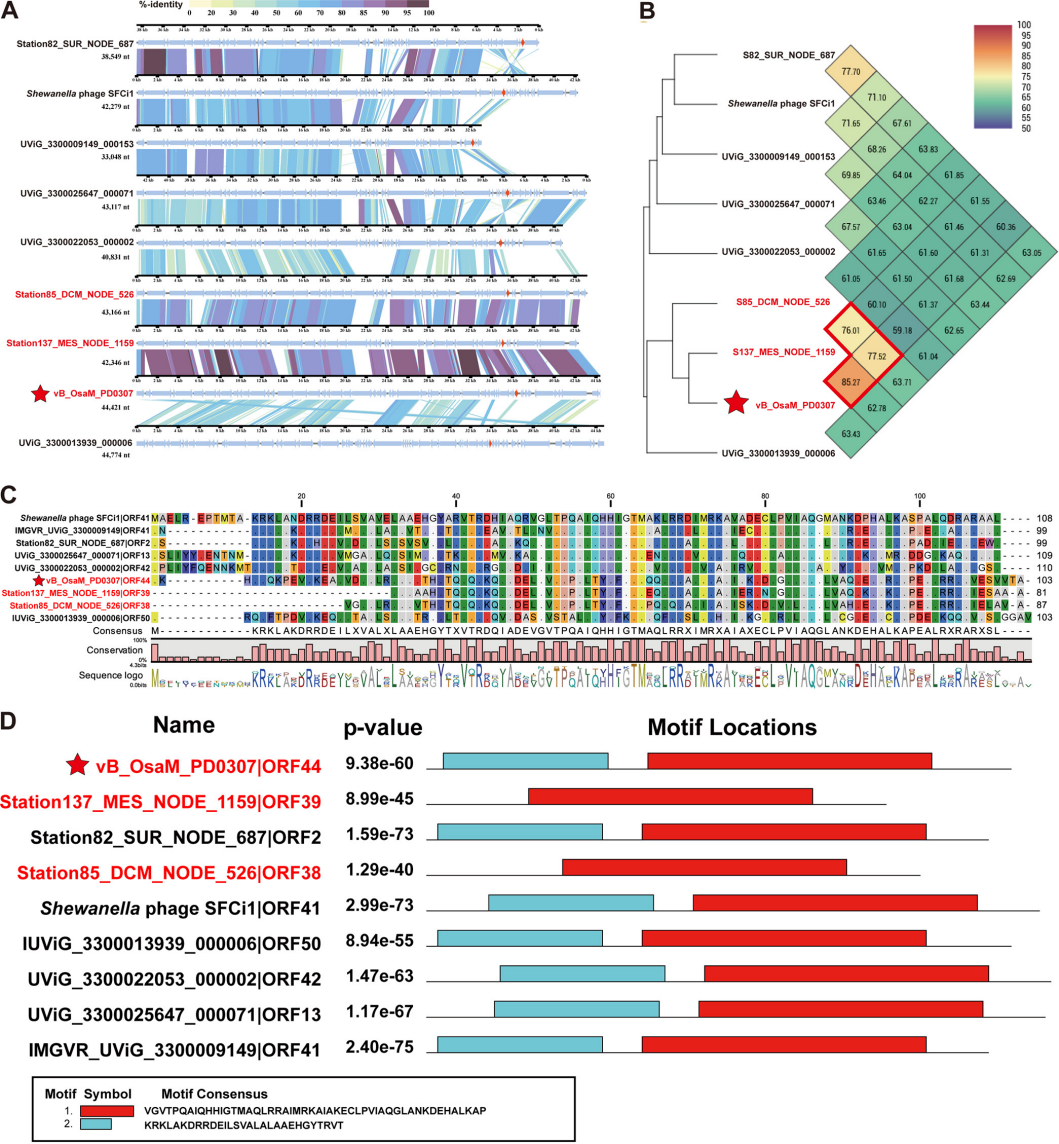
The synteny, ANI, and *TetR* motif of *Oceanospimyovirus* and six vB_OsaM_PD0307-type sequences. These sequences are on the same branch as vB_OsaM_PD0307 in the VICTOR tree and at the species level, according to 95% ANI. (A) Alignment and comparison of *Oceanospimyovirus* and six vB_OsaM_PD0307-type genomes. *Oceanospimyovirus* are shown in red. The arrows filled in red indicate the AMG-*TetR* gene. The color of the shading connecting homologous genes indicates the level of amino acid identity between the genes. (B) The ANIs of *Oceanospimyovirus* and vB_OsaM_PD0307-type genomes, based on OrthoANI values that were calculated using OAT software. The ANIs of three *Oceanospimyovirus* are more than 70% in the red box. (C) Alignment of nine TetR genes in the nine viruses. The consensus, conservation, and sequence logo show the evolutionary conservation of each amino acid, based on alignment. (D) The *P* values and the locations of two motifs in nine *TetR* protein sequences. The motif consensus is shown at the bottom. Extremely low *P* values indicate that the motifs were reliable.

To clarify the common features of *Oceanospimyovirus* and the homologous viral genomes (vB_OsaM_PD0307-type viruses), an alignment of these sequences via tBLASTx was performed. Eight viral genomes at the species-level (≥95% nucleotide identity) were on the same branch as vB_OsaM_PD0307 in the phylogenetic tree that was constructed using VICTOR (Fig. 3), and these were selected for a comparative genomic analysis (55). The genomic analysis confirmed that *Oceanospimyovirus* exhibits genomic synteny with vB_OsaM_PD0307-type viruses (Fig. 5A). Although some of these vB_OsaM_PD0307-type viruses have genomic variations, most viruses appear to be highly similar to *Oceanospimyovirus*.

It is worth noting that the AMG of vB_OsaM_PD0307, namely, the *TetR* family transcriptional regulator gene, was found in all of the other eight viral genomes (Fig. 5A). The AMG is homologous and has a similar position in the genome’s arrangement (shown with red arrows). All nine *TetR* genes have the same conserved domain, namely, TetR_N (pfam00440), and two consensus motifs that were identified via MEME and CLC Genomic Workbench (Qiagen, Hilden, Germany) (Fig. 5C and D) (56). The sequence analysis revealed that all three genomes of *Oceanospimyovirus* and six vB_OsaM_PD0307-type viruses contained this unusual *TetR* protein domain, which might be an important genomic feature of the *Oceanospimyovirus* genus.

### Biogeographic distributions of *Oceanospimyovirus* in the global ocean.

To identify the relative abundance and distribution patterns of *Oceanospimyovirus* and the vB_OsaM_PD0307-type genome, a metagenomic read recruitment was performed by mapping reads from 154 viral metagenomes; these were divided into 5 viral ecological zones (VEZs) of the Global Ocean Viromes (GOV2.0) data set: Arctic (ARC), Antarctic (ANT), bathypelagic (BATHY), temperate and tropical epipelagic (EPI), and temperate and tropical mesopelagic (MES) (57, 58).

**FIG 6:**
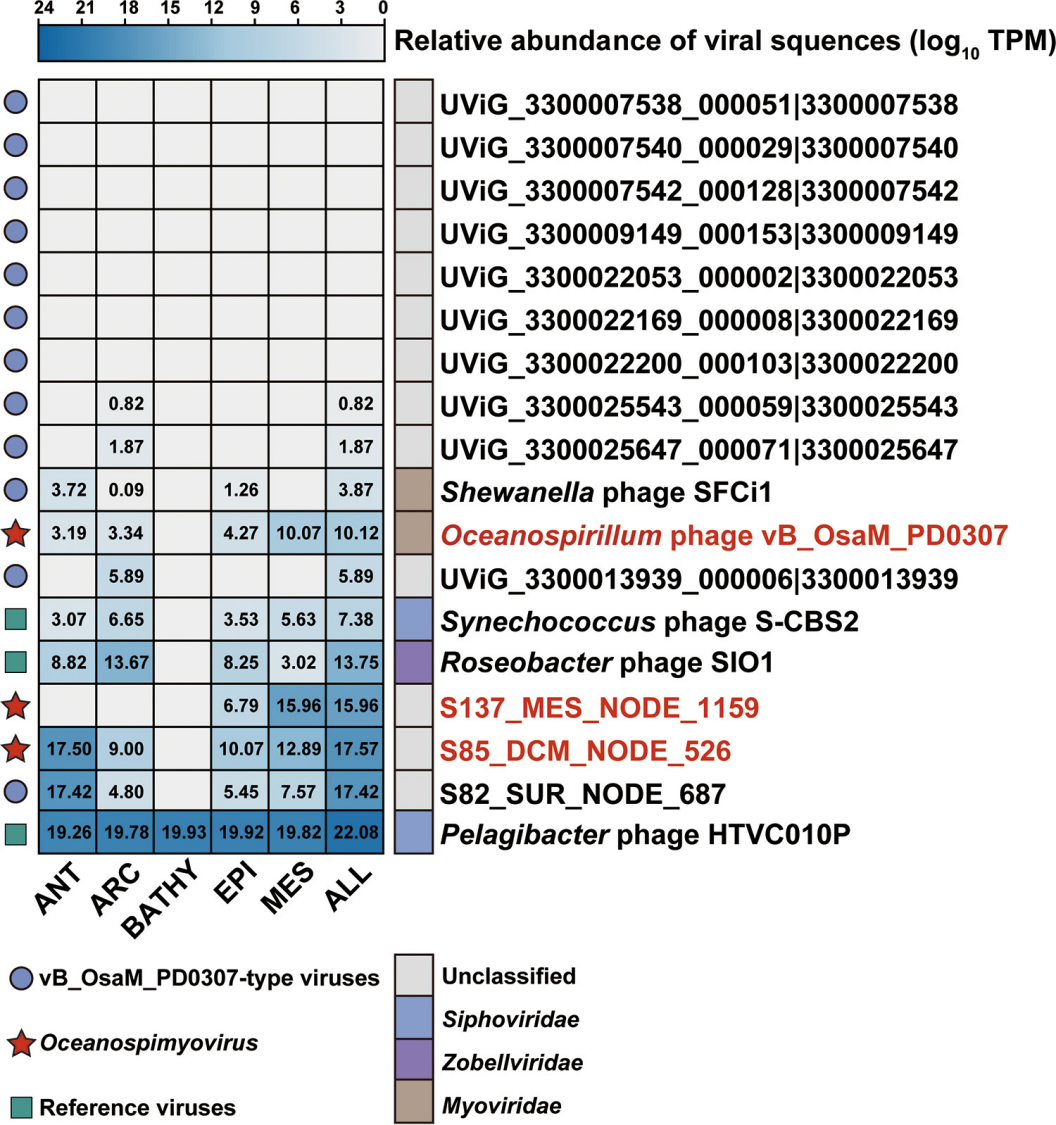
Heatmap displaying the relative abundance of *Oceanospimyovirus* and vB_OsaM_PD0307-type phages in the global ocean viromes (GOV 2.0). The normalized relative abundance is depicted in terms of TPM (transcripts per kilobase of exon model per million mapped reads) and was log_10_ transformed for description using the metagenomics tool minimap2. Representative Pelagibacter phage HTVC010P, Synechococcus phage S-CBS2, and roseophage SIO1 were used as references to calculate the abundances.

Viromic reads mapped to *Oceanospimyovirus* were present in all EPI and MES viromes (0 to 1,000 m) with various relative abundances (Fig. 6). In contrast, neither genome was detected in BATHY viromes (>1,000 m). This observation suggests that the hosts infected by these phages displayed a limited distribution and might not be able to adapt to deep-sea environments. It was also noticed that the abundance of S85_DCM_NODE_526 in ANT was significantly different from the other *Oceanospimyovirus*, suggesting that the most abundant members in this genus have not yet been isolated. The vB_OsaM_PD0307-type member, namely, S82_SUR_NODE_687, was also frequently detected at polar stations and has a similar distribution pattern to S85_DCM_NODE_526 (Fig. 6). Furthermore, some phages were found to be abundant in polar areas, whereas others were prevalent in nonpolar stations, suggesting that they may infect hosts with broader distribution and could infect either cold-type or warm-type hosts. These results show that S85_DCM_NODE_526 is common in the Southern Ocean, that *Oceanospimyovirus* is abundant in the polar regions, and that vB_OsaM_PD0307-type viruses have biologically and ecologically important distributions in the global ocean.

### Conclusion.

*Oceanospirillaceae* has a strong metabolic capacity and occupies a key ecological niche. As such, its phage will inevitably affect the abundance of hosts as well as their community structure and metabolic capacity. Here, we isolated the first myovirus against *Oceanospirillaceae*, named vB_OsaM_PD0307, which represents a novel genus-level cluster, named *Oceanospimyovirus*, with two high-quality UViGs. *Oceanospimyovirus* carried the same antibiotic resistance gene-*TetR* family transcriptional regulator in their genomes. The relative abundance of *Oceanospimyovirus* suggests that it is an abundant viral genus in polar regions and plays an important ecological role in the global ocean. These novel insights into the diversity and ecology of *Oceanospimyovirus* further expand our current understanding of these important phages.

## MATERIALS AND METHODS

### Ethics statement.

This article does not contain any studies with animals or human participants that were performed by any of the authors.

### Bacteria and phage isolation.

Both *Oceanospirillum* sp. PD0307 and its phage vB_OsaM_PD0307 were isolated from a sample that was collected from coastal surface waters of the Yellow Sea (120°19’32.6“E, 36°4’1.7”N) in June of 2020. The host strain was isolated from and maintained in 2216E medium (peptone 5 wt.%, yeast extract 1 wt.%) at 28°C and 120 rpm in a shaking incubator.

A total of 355 16S rRNA sequences of *Oceanospirillaceae* with defined taxonomy in GenBank, including that of the host strain *Oceanospirillum* sp. PD0307, were retrieved from GenBank and were aligned via MAFFT, using the G-INS-1 strategy with 1,000 iterations (59). The maximum likelihood phylogenic tree was calculated from multiple sequence alignments using IQ-tree2 (60), applying the GTR+I+G model with 1,000 bootstrap iterations, and it was visualized using iTOL v5 (61).

For phage isolation, 10 mL seawater samples collected from the same station were passed through a 0.22 μm membrane filter to remove any cellular organisms (Isopore 0.2 μm GTTP, Merck, Ireland). Phage vB_OsaM_PD0307 was isolated via plaque assay using the double-layer plating method (62). Briefly, 200 μL seawater were filtered through 0.22 μm pore-size filters and were mixed with the host culture (for approximately 8 h) and incubated for 30 minutes, thereby allowing for the adsorption of the phages at room temperature. Then, 4 mL of the semisolid culture (at 55°C) was added to the mixture and poured onto the plate after vortexing. The plates were cultivated at 28°C and monitored until visible plaques were formed in the double layer culture, which usually happens within 24 h.

### Phage purification and morphology.

A single plaque was picked from the double plate, suspended in 2 mL of SM buffer, (100 mM NaCl, 8 mM MgSO_4_, 50 mM Tris-HCl, at pH 7.5) and then purified three times via plaque assay. Culture lysates were recovered to enrich and concentrate the phages. Approximately 500 mL of exponentially growing host was incubated with 50 mL of purified viral stock and were incubated at 28°C for 24 h. The lysate was first filtered through a 0.22 μm membrane filter to remove uninfected host cells, and the lysate was then concentrated from 500 mL to 5 mL using 30 kDa superfilters (UFC5030, Millipore). The concentrated phage lysate was treated with a second filtration by passing through a 0.22 μm Supor membrane.

The supernatant solution was precipitated by adding PEG 8000 and NaCl to a final concentration of 10% (wt/vol) and 1 M, respectively, and it was incubated overnight at 4°C. After precipitation, the pellet was centrifuged at 15,000 × *g* for 30 min and then resuspended in 5 mL of SM buffer (63). The purified phage solution was stored away from light at 4°C.

The morphology of vB_OliS_GJ44 was analyzed via transmission electron microscopy (TEM), using the established protocols of the negative staining method (64). 20 μL of the mixture were then taken, placed on a copper net, and stained with 2 wt.% phosphotungstic acids (pH 7.5) for 5 min. Its morphology was identified using a TEM (JEOLJEM-1200EX, Japan) at 100 kV that was equipped with a diamond knife for thin sectioning. Images were taken using a GATAN INC CCD image transmission system (Gatan Inc., Pleasanton, CA, USA).

### Phage genome sequencing and bioinformatics analysis.

DNA was extracted from the concentrated phage lysate using a Virus DNA Kit (Omega Bio-tek, GA, USA), according to the manufacturer’s instructions. Quality control was subsequently carried out on the purified DNA samples. The high-quality DNA sample (OD260/280 = approximately 1.8 to 2.0, >6 μg) was used to construct the fragment library and was then used for Illumina NovaSeq 6000 sequencing by Shanghai Biozeron Biotechnology Co., Ltd. (Shanghai, China). The raw paired-end reads were trimmed and quality controlled using Trimmomatic (v. 0.3.6) with the following parameters: SLIDINGWINDOW: 4:15, MINLEN: 75 (65). ABySS was used to assemble the viral genome after the quality control processes, and multiple-Kmer parameters were chosen to obtain the optimal assembly results (66). GapCloser software was subsequently applied to fill in the remaining local inner gaps and to correct the single base polymorphism for the final assembly and further analysis (67).

ORFs were initially predicted using GeneMarkS and annotated using BLASTp against the nonredundant (NR), Swiss-Prot (http://uniprot.org), KEGG (http://www.genome.jp/kegg/), and COG (http://www.ncbi.nlm.nih.gov/COG) databases (68). The genome visualization was conducted using CLC Main Workbench (Qiagen, Hilden, Germany). A GC skew analysis was performed on Genskew (https://genskew.csb.univie.ac.at/webskew). The termini positions of the phage genome were determined using PhageTerm, which uses the raw reads of a phage that was sequenced with a sequencing technology via random fragmentation and its genomic reference sequence (30).

### Network analysis.

vConTACT 2.0 is a network-based application that utilizes whole-genome gene-sharing profiles for virus taxonomic predictions (53). It replicated near-identical (96%) existing genus-level viral taxonomy assignments from the International Committee on Taxonomy of Viruses for National Center for Biotechnology Information virus RefSeq. Viral clusters (VCs) were defined using ClusterONE, rather than the Markov cluster algorithm, which was used in vConTACT v.1.0. Four main parameters of ClusterONE were used to detect the complex network relationships and format the different VCs, density (≥0.3), node penalty (=2.0), haircut (=0.65), and overlap (=0.8). The density and node penalty parameters represent the cohesiveness of a cluster, the haircut parameter represents the boundaries of the clusters (that is, outlier genomes), and the overlap parameter represents the size of the overlap between clusters (53). To describe the taxonomy of vB_OsaM_PD0307 in detail, a total of 14,118 isolated complete Caudovirales genomes were downloaded from the NCBI Virus database to build a reference data set. At the same time, all of the protein sequences of vB_OsaM_PD0307 were queried against the whole IMG/VR protein database, which included 2,314,329 UViGs, using BLASTp with an E value of <1×10^−5^ to recruit as many homologous sequences as possible. Finally, 8,799 uncultivated viral genomes that had at least two proteins that were homologous with vB_OsaM_PD0307 were selected and added to the reference data set to construct the first network using vConTACT (53). For better presentation, we selected 595 viral sequences that were related to vB_OsaM_PD0307, according to the weight (>30) and taxonomic information (belonging to the same subfamily as vB_OsaM_PD0307) that were generated using vConTACT and visualized using Gephi (53, 69).

### Phylogenetic and synteny analysis.

Phylogenetic trees were conducted to evaluate the evolutionary relationship and taxon classification of vB_OsaM_PD0307 via three different methods.

(i) A “proteomic tree” of vB_OsaM_PD0307 and all reference viral genomes in the Virus-Host DB, based on genome-wide sequence similarities that were computed using tBLASTx, was generated using ViPtree (50). For visualization purposes, only queries and genomes that were related to the query viruses are shown (*S_G_* > 0.02).

(ii) A phylogenetic tree of terminase large subunits (TerL) domains was created, based on the results of vConTACT (53). ORFs of all of the 595 vB_OsaM_PD0307-related viral genomes were searched against the PfamA database with HMMER for recognizable conserved TerL domains (70). Manual inspection ensured that at most one TerL was selected for each viral genome sequence. Then, 391 TerL domains were candidates. To collect reference sequences, four TerL domain HMM models (PF05876 Terminase_GPA, PF03354 TerL_ATPase, PF03237 Terminase_6N, and PF04466 Terminase_3) of the PfamA seed database were downloaded and compressed. The TerL domain HMM models were searched for phage large-subunit-terminase, domain-containing proteins of all of the complete and family-taxon Caudophages in the GenBank, and these domain sequences were extracted from the proteins and were clustered as the final reference sequence, using CD-HIT to remove redundancy (71). The final set contained 1,161 TerL domain sequences. These were aligned using MAFFT. The maximum likelihood phylogenetic tree was computed using IQ-tree2 with 1,000 bootstrap replicates and the Q.pfam+F+R10 model, and it was visualized using iTOL (61).

(iii) According to the weight results that were calculated using vConTACT, the top 100 most associated with vB_OsaM_PD0307 (weight > 40, genome completeness > 50%) were selected for the construction of a whole-genome phylogenomic tree that was inferred using the Genome-BLAST Distance Phylogeny method (GBDP), with suggestions for classification at the species, genus, subfamily, and family levels via VICTOR, and the tree was visualized using iTOL (55).

14 viruses that were on the same clades with vB_OsaM_PD0307 in the VICTOR-tree were clustered with 95% nucleotide identity by CD-HIT (71). Finally, nine viruses were combined to perform synteny using ViPtree and to calculate the intergenomic ANI via OAT, using the orthogonal method to determine the overall similarity (50, 72).

The genome completeness of the UViGs was assessed using CheckV (73), and the virulence possibility was predicted using BACPHLIP (29). The sample resources, genome topology, taxon information, host prediction, and other information of the UViGs were collected from the IMG/VR database (52). The motif features of the *TetR* protein sequence were represented using CLC Genomics Workbench (Qiagen, Hilden, Germany) and the MEME Suite web server (56).

### Biogeographic distributions in the oceans.

The relative abundance of *Oceanospimyovirus* phages in marine viromes was estimated through a viromic read-mapping analysis. The Global Oceans Viromes (GOV 2.0) data set was downloaded for access to the relative abundance values (7). All 154 stations were divided into 5 viral ecological zones (VEZs), including the Arctic (ARC), Antarctic (ANT), temperate and tropical epipelagic (EPI), temperate and tropical mesopelagic (MES), and bathypelagic (BATHY) zones. The relative abundance in the global ocean metagenomes was calculated by using the metagenomics tool minimap2 (identity of 0.95, aligned percent of 0.75) (74). Additionally, pelagiphage HTVC010P, *Synechococcus* phage S-CBS2, and roseophage SIO1 are significantly representative and have other 12 viruses on the same branch with *Oceanospimyovirus* in the VICTOR-tree as the references.

### Data availability.

The complete genome of *Oceanospirillum* phage vB_OsaM_PD0307 has been deposited in the NCBI GenBank under accession number OL658619, and the 16S rRNA sequence of the host has also been deposited in the NCBI GenBank under accession number OL636378.
