# Supplementary material for: Discovery of an Abundant Viral Genus in Polar Regions through the Isolation and Genomic Characterization of a New Virus against Oceanospirillaceae
Source: Appl Environ Microbiol. 2023 Mar 28;89(4):e01896-22. doi: 10.1128/aem.01896-22 (PMC10132091; doi:10.1128/aem.01896-22)
Supplement: Supplemental file 1 — Supplemental material. Download aem.01896-22-s0001.pdf, PDF file, 5.8 MB [file aem.01896-22-s0001.pdf]

SUPPLEMENTAL MATERIALS

Fig. S1 The maximum likelihood phylogenic tree of *Oceanospirillum* sp. PD0307 and 16S rRNA sequences of 31 related *Oceanospirillaceae* species. The *Oceanospirillum* sp. PD0307 was highlighted in the tree. A relatively close relationship between *Oceanospirillum* sp. PD0307 with other *Oceanospirillum* was displayed, while monophyly of its branch was observed.

Fig. S2 PhageTerm Analysis report of Oceanospirillum phage vB_OsaM_PD0307.

Fig. S3 Phylogenetic tree (A), ANI (B) and synteny analysis (C) between Oceanospirillum phage vB_OsaM_PD0307 and other phages against *Oceanospirillaceae.*

Fig. S4 The circular viral proteomic tree of vB_OsaM_PD0307 with 1,824 dsDNA phage isolates in the Virus-Host database as references constructed by ViPtree.

Fig. S5 Gene-sharing network of vB_OsaM_PD0307 and related viral sequences. The network was conducted with the Caudovirales from the NCBI RefSeq database and the vB_OsaM_PD0307 related-UViGs from IMG/VR database. we only showed the nodes which have a weight > 30 with vB_OsaM_PD0307 and removed the singleton nodes for better visualization. The nodes represent the viral genomes. The edges represent the weight between viral genomes based on shared gene content. VC is the abbreviation of viral cluster produced from vConTACT. The nodes framed by pentagrams are the members of *Oceanomyovirus* genus, and the red is vB_OsaM_PD0307, yellow is S137_MES_NODE_1159 and pink is

S85_DCM_NODE_526, respectively. (A) colored base on the VCs generated by vConTACT (B) colored based on the taxon information of viuses (C) colored based on the resources of viral sequences.

Table S1 Genome annotation of Oceanospirillum phage vB_OsaM_PD0307

Table S2 HHperd hits of Oceanospirillum phage vB_OsaM_PD0307

Fig. S1

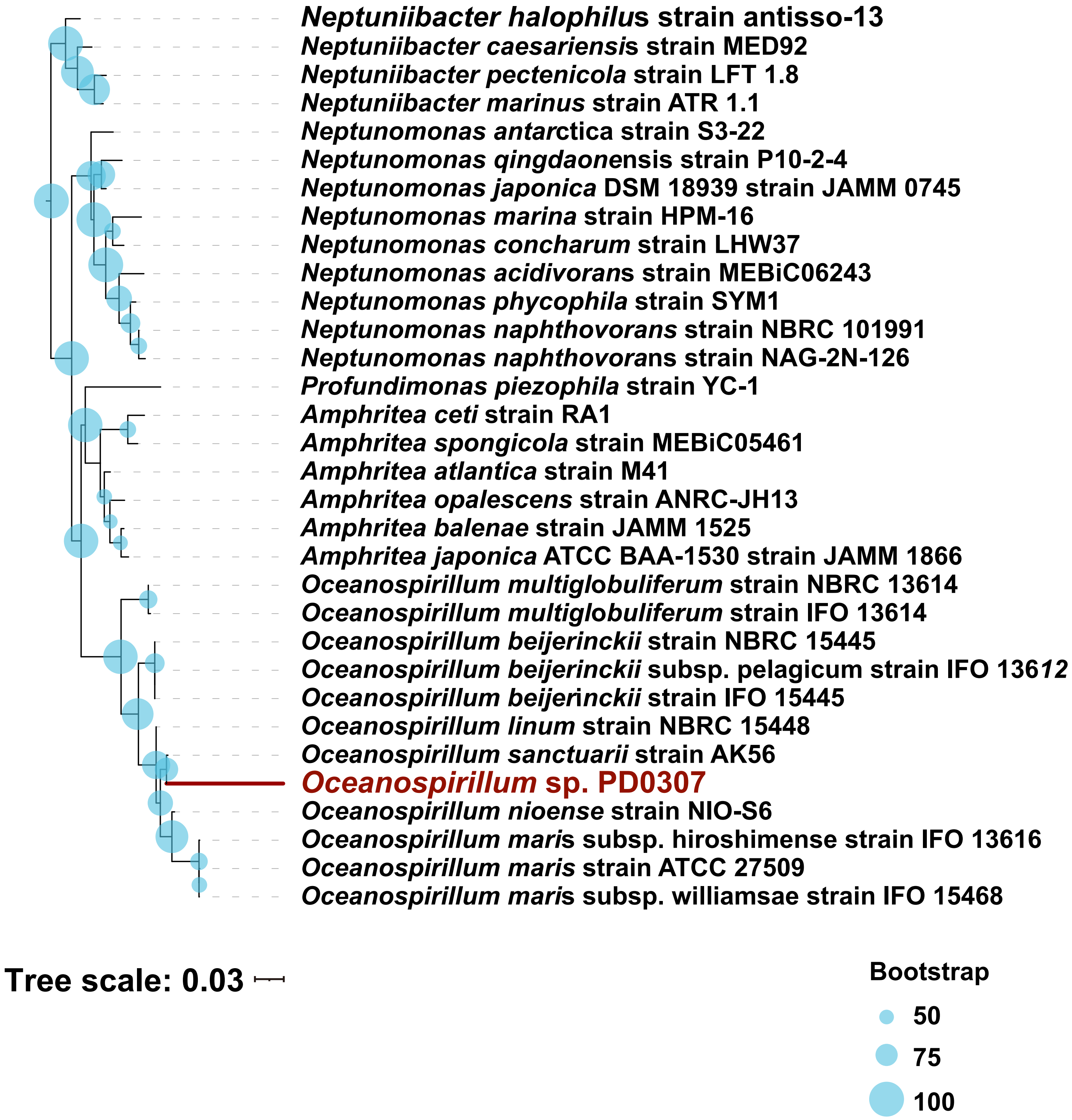

Neptuniibacter halophilus strain antisso-13
Neptuniibacter caesariensis strain MED92
Neptuniibacter pectenicola strain LFT 1.8
Neptuniibacter marinus strain ATR 1.1
Neptunomonas antarctica strain S3-22
Neptunomonas qingdaonensis strain P10-2-4
Neptunomonas japonica DSM 18939 strain JAMM 0745
Neptunomonas marina strain HPM-16
Neptunomonas concharum strain LHW37
Neptunomonas acidivorans strain MEBiC06243
Neptunomonas phycophila strain SYM1
Neptunomonas naphthovorans strain NBRC 101991
Neptunomonas naphthovorans strain NAG-2N-126
Profundimonas piezophila strain YC-1
Amphritea ceti strain RA1
Amphritea spongicola strain MEBiC05461
Amphritea atlantica strain M41
Amphritea opalescens strain ANRC-JH13
Amphritea balenae strain JAMM 1525
Amphritea japonica ATCC BAA-1530 strain JAMM 1866
Oceanospirillum multiglobuliferum strain NBRC 13614
Oceanospirillum multiglobuliferum strain IFO 13614
Oceanospirillum beijerinckii strain NBRC 15445
Oceanospirillum beijerinckii subsp. pelagicum strain IFO 13612
Oceanospirillum beijerinckii strain IFO 15445
Oceanospirillum linum strain NBRC 15448
Oceanospirillum sanctuarii strain AK56
Oceanospirillum sp. PD0307
Oceanospirillum nioense strain NIO-S6
Oceanospirillum maris subsp. hiroshimense strain IFO 13616
Oceanospirillum maris strain ATCC 27509
Oceanospirillum maris subsp. williamsae strain IFO 15468
Tree scale: 0.03
Bootstrap
50
75
100

Fig. S2

Oceanospirillum phage vB_OsaM_PD0307 PhageTerm Analysis

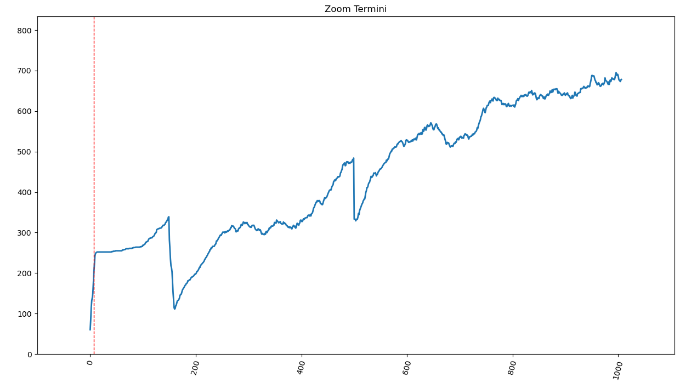

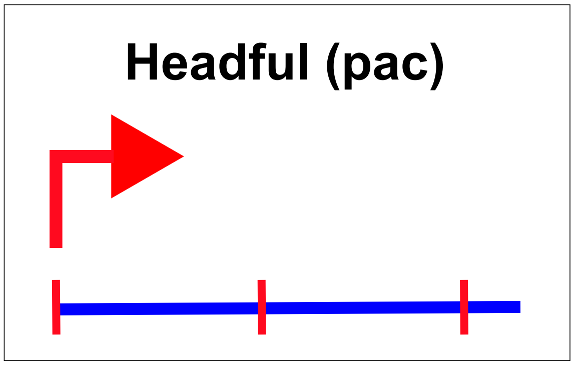

PhageTerm Method

Ends	Left (red)	Right (green)	Permuted	Orientation	Class	Type
Redundant	7	Distributed	Yes	Forward	Headful (pac)	P1

Strand	Location	T	pvalue
+	7	0.46	1.03e-05
	12905	0.05	1.00e+00
	22386	0.05	1.00e+00
	43448	0.04	1.00e+00
	19676	0.04	1.00e+00
-	35237	0.05	1.00e+00
	43126	0.05	1.00e+00
	31504	0.05	1.00e+00
	24334	0.04	1.00e+00
	42344	0.04	1.00e+00

T (Start. Pos. Cov. / Whole Cov.)

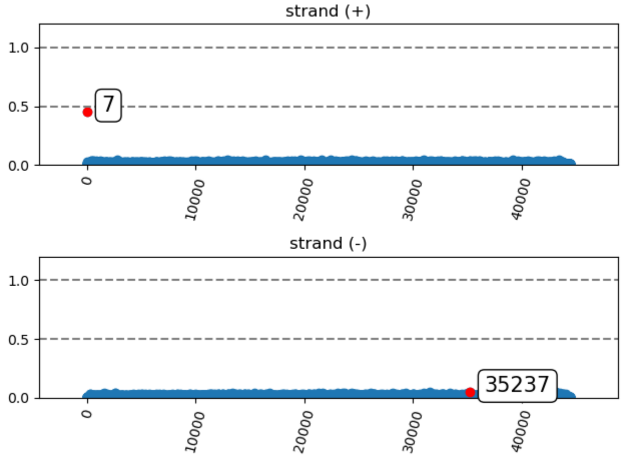

Analysis Methodology

PhageTerm software uses raw reads of a phage sequenced with a sequencing technology using random fragmentation and its genomic reference sequence to determine the termini position. The process starts with the alignment of NGS reads to the phage genome in order to calculate the starting position coverage (SPC), where a hit is given only to the position of the first base in a successfully aligned read (the alignment algorithm uses the lenght of the seed (default: 20) for mapping and does not accept gap or missmatch to speed up the process). Then the program apply 2 distinct scoring methods: i) a statistical approach based on the Gamma law; and ii) a method derived from LI and al. 2014 paper.

General set-up and mapping informations

**Phage Genome**	44421 bp
**Sequencing Reads**	913039
**Mapping Reads**	97 %
OPTIONS	
Mapping Seed	*20*
Surrounding	*20*
Host Analysis	*No*

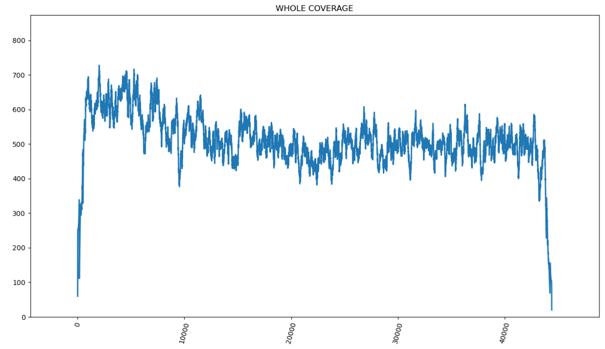

Highest peak of each side coverage graphics

Whole Coverage Zoom (Left) | **Whole Coverage Zoom (Right)**

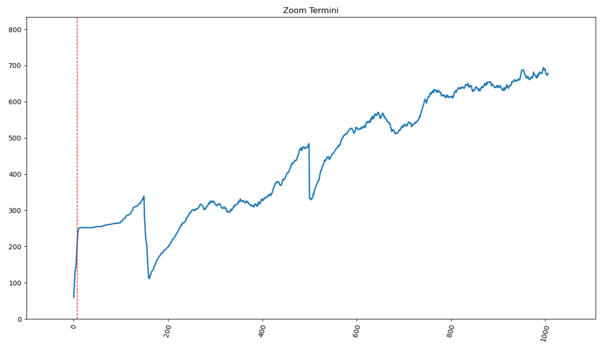

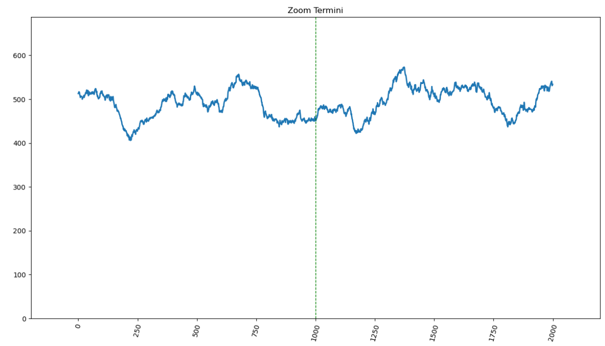

General controls information

**Whole genome coverage**	250	OK
**Weak genome coverage**	0.0 %	OK
**Reads lost during alignment**	2.9 %	OK

PhageTerm method

Reads are mapped on the reference to determine the starting position coverage (SPC) as well as the coverage (COV) in each orientation. These values are then used to compute the variable T = SPC/COV. The average value of T at positions along the genome that are not termini is expected to be 1/F, where F is the average fragment size. For the termini that depends of the packaging mode. Cos Phages: no reads should start before the terminus and therefore T=1. DTR phages: for N phages present in the sample, there should be N fragments that start at the terminus and N fragments that cover the edge of the repeat on the other side of the genome as a results T is expected to be 0.5. Pac phages: for N phages in the sample, there should be N/C fragments starting at the pac site, where C is the number of phage genome copies per concatemer. In the same sample N fragments should cover the pac site position, T is expected to be (N/C)/(N+N/C) = 1/(1+C). To assess whether the number of reads starting at a given position along the genome can be considered a significant outlier, PhageTerm first segments the genome according to coverage using a regression tree. A gamma distribution is fitted to SPC for each segment and an adjusted p-value is computed for each position. If several significant peaks are detected within a small sequence window (default: 20bp), their X values are merged.

**Nearby Termini (Forward / Reverse)**	3 / 0	Peaks localized 20 bases around the maximum

Fig. S3

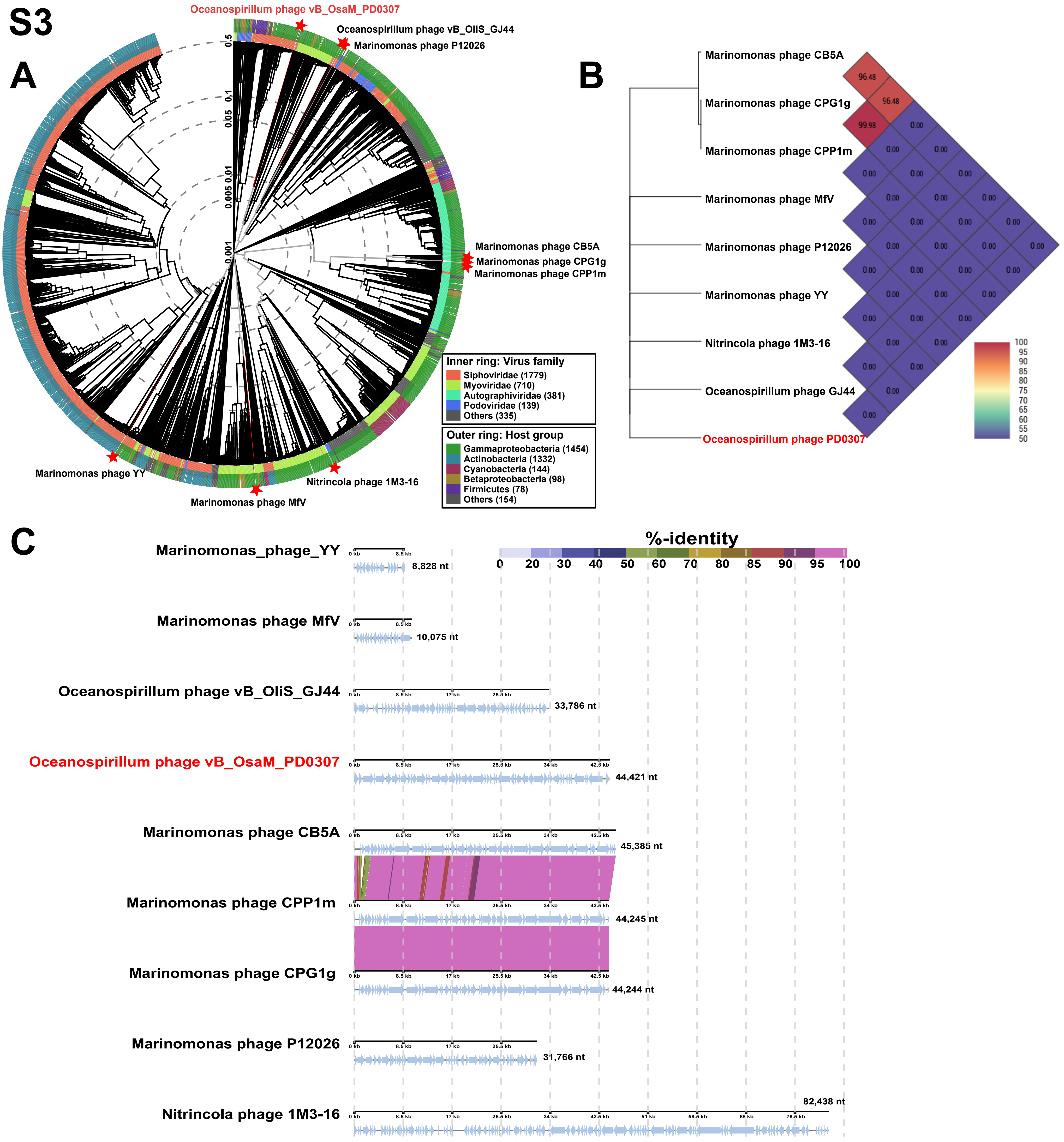
A
Oceanospirillum phage vB_OsaM_PD0307
Oceanospirillum phage vB_OliS_GJ44
Marinomonas phage P12026
Marinomonas phage CB5A
Marinomonas phage CPG1g
Marinomonas phage CPP1m
Marinomonas phage YY
Marinomonas phage MfV
Nitrincola phage 1M3-16
0.5
0.1
0.05
0.01
0.005
0.001
Inner ring: Virus family
Siphoviridae (1779)
Myoviridae (710)
Autographiviridae (381)
Podoviridae (139)
Others (335)
Outer ring: Host group
Gammaproteobacteria (1454)
Actinobacteria (1332)
Cyanobacteria (144)
Betaproteobacteria (98)
Firmicutes (78)
Others (154)
B
Marinomonas phage CB5A
Marinomonas phage CPG1g
Marinomonas phage CPP1m
Marinomonas phage MfV
Marinomonas phage P12026
Marinomonas phage YY
Nitrincola phage 1M3-16
Oceanospirillum phage GJ44
Oceanospirillum phage PD0307
96.48
96.48
99.98
0.00
100
95
90
85
80
75
70
65
60
55
50
C
%-identity
0 20 30 40 50 60 70 80 85 90 95 100
Marinomonas_phage_YY
8,828 nt
Marinomonas phage MfV
10,075 nt
Oceanospirillum phage vB_OliS_GJ44
33,786 nt
Oceanospirillum phage vB_OsaM_PD0307
44,421 nt
Marinomonas phage CB5A
45,385 nt
Marinomonas phage CPP1m
44,245 nt
Marinomonas phage CPG1g
44,244 nt
Marinomonas phage P12026
31,766 nt
Nitrincola phage 1M3-16
82,438 nt
0 kb
8.5 kb
17 kb
25.5 kb
34 kb
42.5 kb
51 kb
59.5 kb
68 kb
76.5 kb

Fig. S4

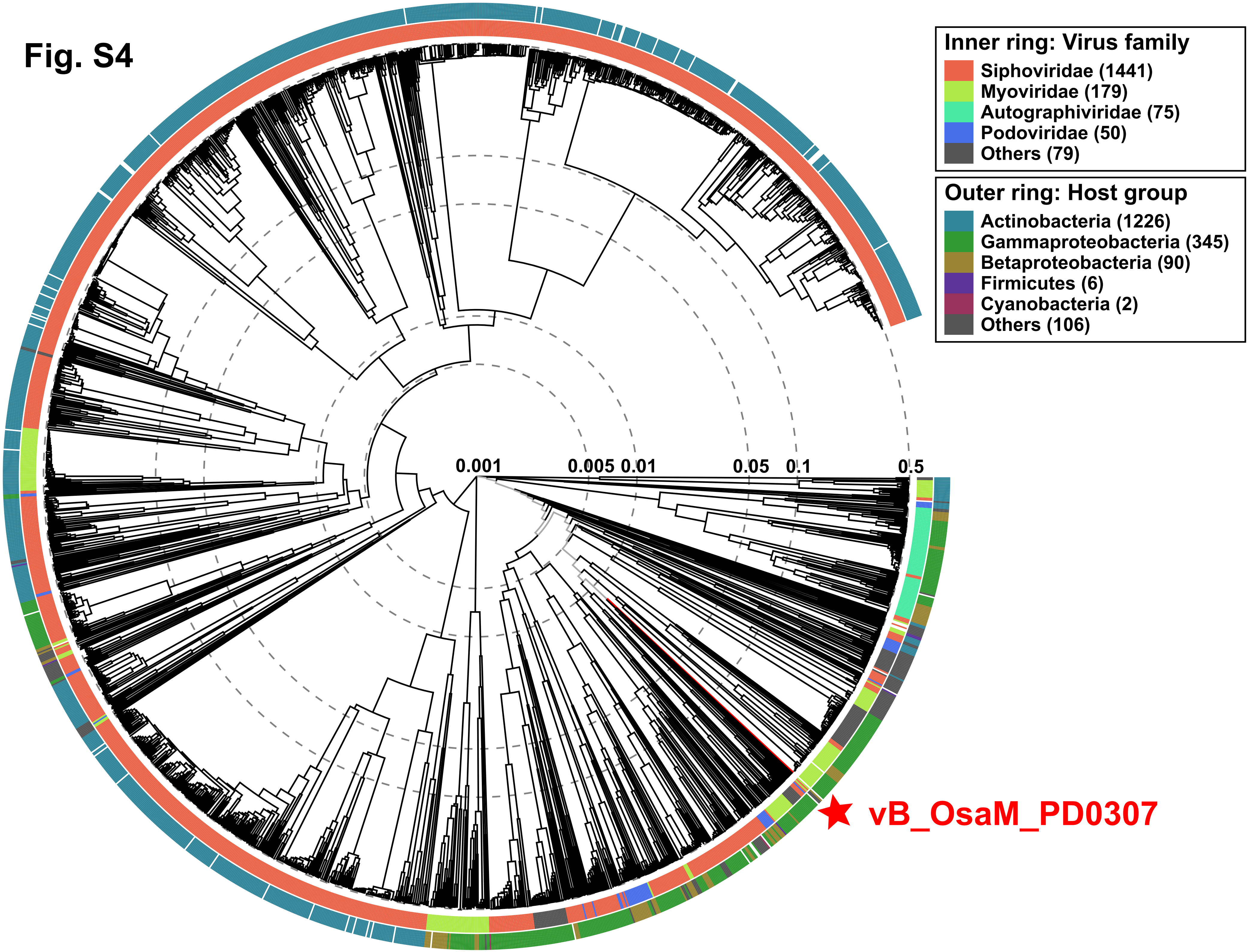
Inner ring: Virus family
Siphoviridae (1441)
Myoviridae (179)
Autographiviridae (75)
Podoviridae (50)
Others (79)
Outer ring: Host group
Actinobacteria (1226)
Gammaproteobacteria (345)
Betaproteobacteria (90)
Firmicutes (6)
Cyanobacteria (2)
Others (106)
0.001
0.005
0.01
0.05
0.1
0.5
vB_OsaM_PD0307

Fig. S5

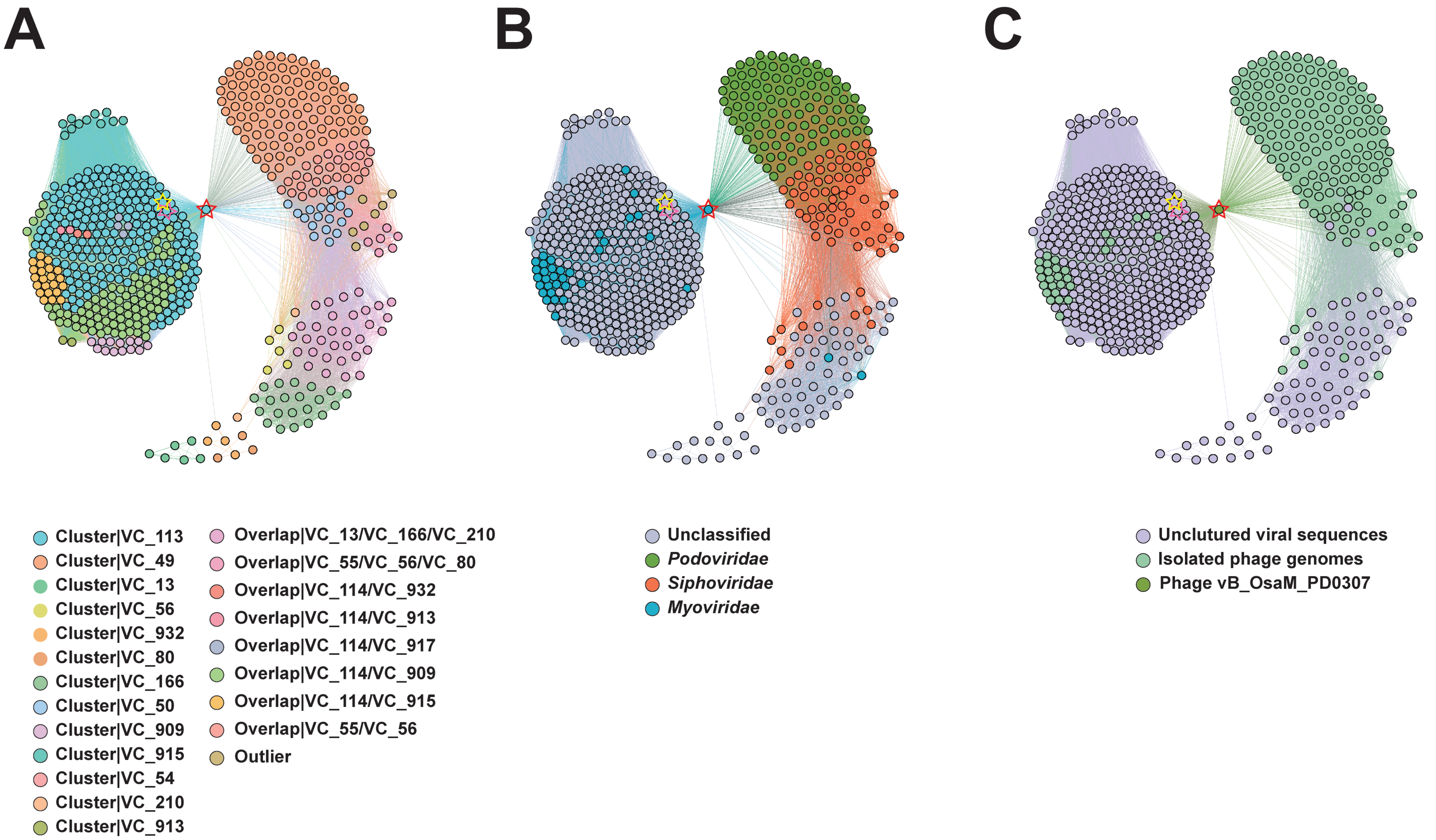
A
B
C
Cluster|VC_113
Cluster|VC_49
Cluster|VC_13
Cluster|VC_56
Cluster|VC_932
Cluster|VC_80
Cluster|VC_166
Cluster|VC_50
Cluster|VC_909
Cluster|VC_915
Cluster|VC_54
Cluster|VC_210
Cluster|VC_913
Overlap|VC_13/VC_166/VC_210
Overlap|VC_55/VC_56/VC_80
Overlap|VC_114/VC_932
Overlap|VC_114/VC_913
Overlap|VC_114/VC_917
Overlap|VC_114/VC_909
Overlap|VC_114/VC_915
Overlap|VC_55/VC_56
Outlier
Unclassified
Podoviridae
Siphoviridae
Myoviridae
Unclutured viral sequences
Isolated phage genomes
Phage vB_OsaM_PD0307

Table S1 Genome annotation of Oceanospirillum phage vB_OsaM_PD0307

Gene	Coding region	Length (aa)	Strand	Start condon	Best hits against nr database	E value	Query coverage (%)	aa Identity (%)	Bitsocre	Conserved domain	Putative function	Module	Methods and databases for searching domins
1	99_779	226	+	ATG	BAV81045.1 hypothetical protein [Vibrio phage CKB-S1]	5.30E-30	99.6	37.8	141.4	P68654 (1.60E-14)	Terminase small subunit	Structure and packaging	Hhpred/UniProtKB
2	772_1440	222	+	ATG	WP_000366434.1 HNH endonuclease [*Salmonella enterica*]	8.00E-39	78.4	47.7	170.6	PF13392 (1.05E-12)	HNH endonuclease	Unclassified	pfam-scan/PfamA
3	1433_3424	663	+	ATG	ANO58028.1 putative large terminase subunit [Shewanella phage SFCi1]	2.00E-266	98.6	64.8	928.3	cl21617 (2.31E-105)	Terminase large subunit	Structure and packaging	Batch CD-Search/CD
4	3485_3748	87	+	TTG	ARB06244.1 hypothetical protein [Shewanella phage SppYZU01]	2.40E-15	96.6	54.8	91.3	P68660 (1.1E-07)	Head completion protein	Structure and packaging	Hhpred-UniProtKB
5	3745_5406	553	+	GTG	ANO58031.1 putative capsid protein [Shewanella phage SFCi1]	3.60E-128	93.9	46.0	468.8	cl19194 (6.51E-49)	Putative capsid protein	Structure and packaging	Batch CD-Search/CD
6	5378_6508	376	+	ATG	ARB06246.1 hypothetical protein [Shewanella phage SppYZU01]	1.70E-65	100.0	43.6	260.0	No hit	Phage portal protein	Structure and packaging	
7	6566_7708	380	+	ATG	ARB06247.1 putative protease [Shewanella phage SppYZU01]	3.50E-103	91.3	57.9	385.2	cl23717 (1.12E-28)	Putative protease	Structure and packaging	Batch CD-Search/CD
8	7713_8114	133	+	ATG	ARB06248.1 hypothetical protein [Shewanella phage SppYZU01]	1.10E-35	100.0	63.2	159.5	P36275 (6.2E-21)	Head decoration protein	Structure and packaging	Hhpred-UniProtKB
9	8127_9215	362	+	ATG	ANO58038.1 hypothetical protein [Shewanella phage SFCi1]	6.80E-120	99.7	57.9	440.7	cl20258 (1.8E-28)	Major capsid protein	Structure and packaging	Batch CD-Search/CD
10	9269_9856	195	+	ATG	ARB06250.1 hypothetical protein	2.10E-06	25.1	67.3	62.8	cl37921 (1.27E-11)	Hypothetical protein	Unclassified	Batch CD-Search/CD
11	9856_10224	122	+	ATG	ARB06251.1 hypothetical protein	3.10E-24	98.4	53.2	121.3	P03714 (7.90E-14)	Head-tail connector protein	Structure and packaging	Hhpred-UniProtKB
12	10221_10916	231	+	ATG	ARB06252.1 hypothetical protein	8.30E-47	98.3	47.8	197.2	No hit	Hypothetical protein	Unclassified	
13	10929_12434	501	+	ATG	ARB06253.1 putative tail protein [Shewanella phage SppYZU01]	1.50E-181	100.0	65.0	646.0	cl26913 (2.9E-40)	Putative tail sheath protein	Structure and packaging	Batch CD-Search/CD
14	12448_12831	127	+	ATG	ARB06254.1 hypothetical protein	3.40E-34	100.0	59.8	154.5	P79679 (3.20E-07)	Tail tube protein	Structure and packaging	Hhpred-UniProtKB
15	12911_13309	132	+	ATG	ARB06255.1 hypothetical protein	5.90E-13	96.2	36.2	84.0	O64313 (2.20E-11)	Tail assembly protein	Structure and packaging	Hhpred-UniProtKB
16	13306_13500	64	+	GTG	No hit					No hit	Hypothetical protein	Unclassified	
17	13505_15130	541	+	ATG	AUR86601.1 TMhelix containing protein [Vibrio phage 1.087.A._10N.261.45.F9]	4.10E-52	98.5	31.0	216.1	P51731 (3.30E-14)	Probable tape measure protein	Structure and packaging	Hhpred-UniProtKB
18	15130_16437	435	+	ATG	ARB06257.1 hypothetical protein	3.20E-116	99.8	52.5	428.7	PF07157 (9.76E-14)	DNA circularization N-terminal domain-containing protein	Structure and packaging	pfam-scan/PfamA
19	16430_17662	410	+	ATG	ARB06258.1 putative tail protein [Shewanella phage SppYZU01]	6.90E-129	97.6	56.5	470.7	cl15796 (6.14E-27)	Putative tail protein	Structure and packaging	Batch CD-Search/CD
20	17653_18207	184	+	GTG	ARB06259.1 hypothetical protein	1.40E-36	95.7	45.3	162.9	Q9T1V4 (1.10E-16)	Putative baseplate protein	Structure and packaging	Hhpred-UniProtKB
21	18207_18641	144	+	ATG	ARB06260.1 hypothetical protein	3.30E-41	97.9	59.6	177.9	Q9T1V3 (7.0E-34)	Baseplate protein	Structure and packaging	Hhpred-UniProtKB
22	18641_19828	395	+	ATG	ARB06261.1 tail protein [Shewanella phage SppYZU01]	4.50E-125	99.7	59.4	458.0	cl01294 (6.68E-34)	Tail protein	Structure and packaging	Batch CD-Search/CD
23	19828_20646	272	+	ATG	ARB06262.1 hypothetical protein	3.40E-84	100.0	59.5	321.6	Q9T1V1 (2.20E-17)	Baseplate protein	Structure and packaging	Hhpred-UniProtKB

24	20659_22608	649	+	ATG	ARB06263.1 hypothetical protein	4.70E-23	25.0	44.2	119.8	PF13229 (8.03E-08)	Putative tail-fiber protein	Structure and packaging	pfam-scan/PfamA
25	22621_23004	127	+	ATG	AUR92108.1 hedgehog signaling/DD-peptidase zinc-binding domain protein<br>[Vibrio phage 1.169.O._10N.261.52.B1]	4.40E-34	100.0	58.6	154.1	PF08291.12 (9.6E-24)	D-Ala-D-Ala carboxypeptidase family metallohydrolase	Lytic	pfam-scan/PfamA
26	23006_23560	184	+	ATG	WP_119396539.1 hypothetical protein<br>[Oceanospirillales bacterium WDS2A16A]	5.30E-44	100.0	51.6	187.6	PF11351.11 (7.30E-5)	TMhelix containing protein	Lytic	Hhpred/PfamA
27	23900_23550	116	-	GTG	WP_004578884.1 hypothetical protein<br>[*Marinobacter nanhaiticus*]	4.90E-04	92.4	29.8	54.3	No hit	Hypothetical protein	Unclassified	
28	24117_23917	66	-	ATG	No hit					No hit	Hypothetical protein	Unclassified	
29	24374_24114	86	-	ATG	No hit					No hit	Hypothetical protein	Unclassified	
30	24838_24371	155	-	ATG	WP_132941300.1 hypothetical protein<br>[*Vibrio crassostreae*]	6.40E-27	54.2	77.4	130.6	No hit	Histidine kinase	Unclassified	
31	27095_24843	750	-	ATG	BAV81076.1 DNA polymerase<br>[Vibrio phage CKB-S1]	2.10E-277	100.0	63.0	964.9	cl02626 (2.34E-88)	DNA polymerase	Nucleotide metabolism	Batch CD-Search/CD
32	27757_27095	220	-	ATG	WP_128316002.1 exonuclease<br>[*Klebsiella pneumoniae*]	1.10E-27	83.6	38.3	133.7	PF16473 (2.37E-44)	Exonuclease	Nucleotide metabolism	pfam-scan/PfamA
33	28043_27816	75	-	ATG	No hit					No hit	Hypothetical protein	Unclassified	
34	28962_28054	302	-	ATG	AAQ96507.1 hypothetical protein<br>[Vibrio phage VP16T]	2.00E-32	57.9	42.2	149.8	4KLK_A (1.6E-29)	Hypothetical protein	Unclassified	Hhpred-PDB
35	30199_29036	387	-	ATG	AUR81608.1 protein of unknown function DUF2800<br>[Vibrio phage 1.009.O._10N.261.51.C9]	8.10E-71	98.7	41.1	277.7	PF10926.9 (3.1E-36)	Exonuclease	Nucleotide metabolism	pfam-scan/PfamA
36	30993_30202	263	-	ATG	AAQ96574.1 hypothetical protein<br>[Vibrio phage VP16C]	1.50E-12	55.5	33.8	83.6	No hit	Hypothetical protein	Unclassified	
37	31082_31240	52	+	ATG	No hit					No hit	Hypothetical protein	Unclassified	
38	31240_32952	570	+	ATG	BAV81083.1 putative helicase<br>[Vibrio phage CKB-S1]	2.00E-195	97.9	61.1	692.2	COG1061 (4.43E-54)	Putative helicase	Nucleotide metabolism	Batch CD-Search/CD
39	32993_33592	199	+	ATG	WP_036406872.1 hypothetical protein<br>[*Morganella morganii*]	6.80E-21	99.0	37.3	110.9	PF18757.2 (5.7E-45)	Nucleotide modification associated protein	Nucleotide metabolism	pfam-scan/PfamA
40	33595_33939	114	+	ATG	WP_114047524.1 DUF4406 domain-containing protein<br>[*Salmonella enterica*]	1.60E-14	82.5	47.9	89.0	cl23749 (3.23E-28)	Nucleoside 2-deoxyribosyltransferase	Nucleotide metabolism	Batch CD-Search/CD
41	33936_34445	169	+	ATG	AAQ96577.1 hypothetical protein<br>[Vibrio phage VP16C]	2.20E-36	97.6	53.6	162.2	4QBL_C (4.10E-15)	Putative nuclease	Nucleotide metabolism	Hhpred-PDB
42	34442_34654	70	+	ATG	No hit					No hit	Hypothetical protein	Unclassified	
43	34651_34845	64	+	ATG	No hit					No hit	Hypothetical protein	Unclassified	
44	34842_35777	311	+	ATG	BAV81086.1 gp155<br>[Vibrio phage CKB-S1]	7.20E-70	88.4	51.1	274.2	cl17173 (2.00E-08)	Site-specific DNA-methyltransferase	Nucleotide metabolism	Batch CD-Search/CD
45	35758_36237	159	+	GTG	BAV81086.1 gp155<br>[Vibrio phage CKB-S1]	2.70E-57	89.9	75.0	231.5	PF01555 (3.63E-16)	Putative DNA methylase	Nucleotide metabolism	pfam-scan/PfamA
46	36255_36566	103	+	ATG	RZJ26198.1 TetR family transcriptional regulator<br>[*Haliea* sp.]	1.60E-10	96.1	43.4	75.5	PF00440 (3.49E-07)	TetR family tanscriptional regulator	AMG	pfam-scan/PfamA
47	36972_36742	76	-	ATG	No hit					No hit	Hypothetical protein	Unclassified	
48	37124_36969	51	-	ATG	AUR95722.1 hypothetical protein NVP1211A_26<br>[Vibrio phage 1.211.A._10N.222.52.F11]	3.30E-04	80.4	61.0	53.5	No hit	Hypothetical protein	Unclassified	

49	37416_37117	99	-	ATG	No hit					PF03333.16 (2.80E-09)	Adhesin biosynthesis transcription regulatory protein	Unclassified	Hhpred-PfamA
50	37796_37413	127	-	ATG	AET42367.1 endonuclease [Emiliania huxleyi virus 202]	9.90E-34	99.2	55.6	152.9	PF03013 (2.23E-34)	Endonuclease	Nucleotide metabolism	pfam-scan/PfamA
51	37954_37796	52	-	ATG	No hit					No hit	Hypothetical protein	Unclassified	
52	39912_38065	615	-	ATG	WP_027591084.1 hypothetical protein [*Pseudomonas* sp. RL]	5.50E-239	99.7	66.0	837.0	cl38938 (9.27E-81)	Putative ribonucleotide reductase	Nucleotide metabolism	Batch CD-Search/CD
53	40355_39909	148	-	ATG	WP_047532890.1 hypothetical protein [*Shewanella* sp. ZOR0012]	5.40E-27	99.2	50.8	130.6	No hit	Hypothetical protein	Unclassified	
54	43103_40611	830	-	ATG	KAB0886876.1 hypothetical protein FZI56_21520 [*Cronobacter sakazakii*]	5.40E-72	50.8	36.6	282.7	cl36938 (4.08E-31)	Putative primase	Nucleotide metabolism	Batch CD-Search/CD
55	43285_43088	65	-	ATG	No hit					PF12728 (7.90E-13)	Putative DNA-binding protein	Nucleotide metabolism	pfam-scan/PfamA
56	43649_44338	229	+	ATG	ARB06240.1 hypothetical protein SppYZU01_03 [Shewanella phage SppYZU01]	1.50E-40	97.4	43.2	176.4	P03731 (7.80E-14)	Neck protein	Structure and packaging	Hhpred/UniProtKB

Table S2 HHpred hits of Oceanospirillum phage vB_OsaM_PD0307

Gene	Hit accession	Description	Probability	E-value	Score	SS	Aligned cols	Target length
1	P68654	TERS_BPP21 Terminase, small subunit OS=Enterobacteria phage P21 OX=10711 GN=1 PE=3 SV=1	99.67	1.6E-14	111.42	16.9	165	182
4	P68660	HCP_LAMBD Head completion protein OS=Escherichia phage lambda OX=10710 GN=W PE=1 SV=1	98.8	1.10E-07	56.95	7.5	64	68
8	P36275	DECO_BPP21 Head decoration protein OS=Enterobacteria phage P21 OX=10711 GN=shp PE=1 SV=1	99.87	6.20E-21	131.02	12.5	113	115
11	P03714	FII_LAMBD Head-tail connector protein FII OS=Escherichia phage lambda OX=10710 GN=FII PE=1 SV=1	99.61	7.9E-14	94.42	13	102	117
14	P79679	TUBE_BPMU Tail tube protein OS=Escherichia phage Mu OX=10677 GN=M PE=1 SV=1	98.81	3.20E-07	67.04	11.7	113	118
15	O64313	TAP_BPP2 Tail assembly protein E OS=Escherichia phage P2 OX=10679 GN=E PE=3 SV=1	99.33	2.20E-11	80.35	8.3	63	91
17	P51731	TMP_BPHC1 Probable tape measure protein OS=Haemophilus phage HP1 (strain HP1c1) OX=1289570 PE=3 SV=1	99.83	3.30E-14	149.73	56	42	689
20	Q9T1V4	BP45_BPMU Baseplate puncturing device gp45 OS=Escherichia phage Mu OX=10677 GN=Mup45 PE=1 SV=1	99.78	1.10E-16	120.54	19.2	148	197
21	Q9T1V3	BP46_BPMU Baseplate protein gp46 OS=Escherichia phage Mu OX=10677 GN=Mup46 PE=1 SV=1	100	7.00E-34	208.3	16.6	138	145

23	Q9T1V1	BP48_BPMU Baseplate protein gp48 OS=Escherichia phage Mu OX=10677 GN=Mup48 PE=1 SV=1	99.76	2.20E-17	141.79	14.1	113	180
25	PF08291.14	Peptidase_M15_3 ; Peptidase M15	99.92	9.60E-24	129.95	11.7	100	102
26	PF11351.11	GTA_holin_3TM Holin of 3TMs, for gene-transfer release	98.23	7.30E-05	52.5	10.7	61	111
34	4KLK_A	Phage-related protein DUF2815 structural genomics, phage-related protein, DUF2815, pfam10991	99.97	1.60E-29	220.76	17.4	169	180
35	PF06023.15	Csa1 CRISPR-associated exonuclease Csa1	99.8	4.70E-18	150.62	17.1	224	281
39	PF18757.4	Nmad5 Nucleotide modification associated domain 5	100	1.60E-46	304.38	23.4	195	204
41	4QBL_C	VRR-NUC Nuclease, HYDROLASE HET: MSE 2.0A {Psychrobacter sp.} SCOP: c.52.1.35	99.64	4.10E-15	115.45	10.5	111	145
49	PF03333.16	Adhesin biosynthesis transcription regulatory protein	99.14	2.80E-09	62.37	9.3	66	93
55	6AMA_L	Putative DNA-binding protein BldC, S. coelicolor, developmental switch, MerR-like, DNA BINDING PROTEIN-DNA complex 3.0	99.42	4.70E-11	43.87	7.7	58	71
56	P03731	COMPL_LAMBD Tail completion protein Z OS=Escherichia phage lambda OX=10710 GN=Z PE=3 SV=1	99.6	7.80E-14	111.05	14.4	185	192
